# The Natural Selection of Herpesviruses and Virus-Specific NK Cell Receptors

**DOI:** 10.3390/v1030362

**Published:** 2009-10-13

**Authors:** Joseph C. Sun, Lewis L. Lanier

**Affiliations:** Department of Microbiology and Immunology and the Cancer Research Institute, University of California, San Francisco, CA 94143, USA

**Keywords:** NK cells, cytomegalovirus, evolution

## Abstract

During the co-evolution of cytomegalovirus (CMV) and natural killer (NK) cells, each has evolved specific tactics in an attempt to prevail. CMV has evolved multiple immune evasion mechanisms to avoid detection by NK cells and other immune cells, leading to chronic infection. Meanwhile, the host has evolved virus-specific receptors to counter these evasion strategies. The natural selection of viral genes and host receptors allows us to observe a unique molecular example of “survival of the fittest”, as virus and immune cells try to out-maneuver one another or for the virus to achieve détente for optimal dissemination in the population.

## Introduction

During the course of evolution, the mammalian immune system has learned to adapt in order to defend the host against invading pathogens. Over time, lymphocytes of the immune system have acquired antigen-specific receptors as part of their arsenal to provide host immune surveillance against foreign substances and combat infectious agents. Some receptors, which arise by gene rearrangement and somatic hypermutation, can be exquisitely specific for processed protein fragments presented on major histocompatibility complex (MHC) molecules (in the case of T cells) or entire pathogen proteins (in the case of B cells and antibodies). Other receptors on innate immune cells, such as dendritic cells and macrophages, are germline-encoded, such as the pattern recognition receptors (PRR), and recognize motifs or conserved molecular patterns present on microorganisms. Natural killer (NK) cells possess many other activating and inhibitory receptors, and specific ligation of these receptors determines whether or not NK cells become activated (reviewed in [1]). Recently, several activating receptors on NK cells have been shown to recognize pathogen components, but only a handful of these receptor-ligand interactions have been characterized. In addition, NK cells possess the activating NKG2D receptor, which recognizes host-encoded MHC class I-related proteins that are expressed on “stressed” cells, including virus-infected and transformed cells[1,2].

Whereas antibodies and B cells can recognize the intact pathogen, T cells require the pathogen components to be processed into short peptide fragments by antigen-presenting cells, and then the primed effector T cells require the pathogen-infected cells to present these pathogen-encoded peptides – MHC complexes to be displayed on the surface of the infected cell for T cell recognition and response. The means by which NK cells recognize certain pathogens has not been entirely elucidated. In mammals, there are different families of receptors on NK cells that serve similar functions. In mice and humans, convergent evolution has resulted in the Ly49 and KIR (Killer cell Immunoglobulin-like Receptor) families of receptors, respectively, both containing inhibitory members that recognize host MHC class I and activating receptors that might detect pathogen-infected cells[1]. To date, the ligands of most of the activating receptors have not been well characterized, but some are thought to be proteins specifically encoded by pathogens. Because we are constantly bombarded with environmental pathogens, there is evidence that selective pressures from virulent microbes have caused the immune system in higher organisms to evolve accordingly. For example, African population studies examining areas historically endemic for malaria demonstrated that *Plasmodium falciparum* infection in humans has been a driving force for the selection of various host immune system genes, such as certain human leukocyte antigen (HLA) alleles, important for protection against infection [3,4].

The selective pressures that drive cells of the immune system to change over time are at work on the pathogen as well. In order to avoid immune detection and survive, viruses have adopted many immune evasion strategies such as blocking expression of molecules (MHC class I and NKG2D ligands) that “announce” the presence of infection or stress, and at the same time viruses carry in their genomes many decoy molecules (such as MHC class I analogs) that can inhibit cells of the immune system from becoming activated (reviewed in [5]). Only by delaying the response mounted by the immune cells or evading the immune system entirely can the virus ensure its own propagation and survival. This review will discuss several known NK cell receptor-pathogen interactions, focusing specifically on cytomegalovirus infections in mouse and human.

## Cytomegaloviruses evade the immune system

The goal of CMV is to avoid elimination by the host immune response so that the virus can persist and be optimally disseminated in the host species population. To achieve this objective CMV, a large double-stranded DNA virus in the β-herpesvirus family, has evolved a large number of genes that prevent recognition of infected cells by the immune system.

**Figure 1.**
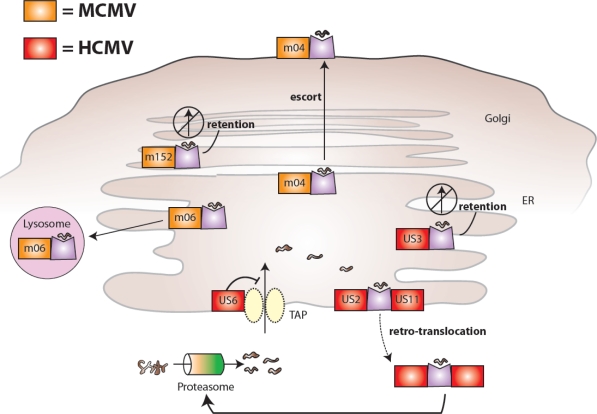
MCMV and HCMV proteins interfere with MHC class I presentation and host recognition of virally infected cells. MCMV-encoded glycoproteins (shown in orange) inhibit the biosynthesis and expression of mouse MHC class I molecules: m06 binds MHC class I in the endoplasmic reticulum (ER) and redirects it to lysosomes leading to degradation, m152 retains MHC class I in an ER-to-Golgi intermediate compartment, and m04 forms a complex with MHC class I in the ER and together they are transported to the cell surface. HCMV-encoded glycoproteins (shown in red) also inhibit the biosynthesis and expression of human MHC class I molecules: US6 prevents translocation of proteasome-generated peptides from entering the ER via TAP (a specific transporter of peptides for loading on MHC class I molecules, associated with antigen presentation), US3 retains MHC class I in the ER, and US2 and US11 both bind MHC class I in the ER and mediate retro-translocation of molecules back to the cytoplasm via the Sec61 channel leading to proteasomal degradation.

During viral infection, CD8^+^ T cells can recognize viral peptides presented on MHC class I and mount a robust response by eliminating infected cells. Thus, CMV would benefit by blocking the pathways leading to host presentation of viral peptides. Many CMV gene products inhibit the presentation of viral proteins on MHC class I [5,6]. The viral proteins m04, m06, and m152 found in MCMV disrupt the MHC class I synthesis pathway (m152 causes MHC class I retention in the pre-Golgi compartment and m06 directs it to the lysosomes for degradation) and modulate antigen presentation (m04 escorts select MHC class I molecules to the cell surface) [7–12]. Similarly, HCMV US2, US3, US6, and US11 downregulate MHC class I presentation at different stages of biosynthesis; US2 and US11 drive retro-translocation of MHC class I from ER to the cytosol leading to degradation, US3 causes its retention in the ER, and US6 inhibits TAP-mediated transport of processed peptides for loading onto MHC class I [13–19]. These MCMV- and HCMV-encoded proteins and their location of function are depicted in Figure 1.

**Figure 2.**
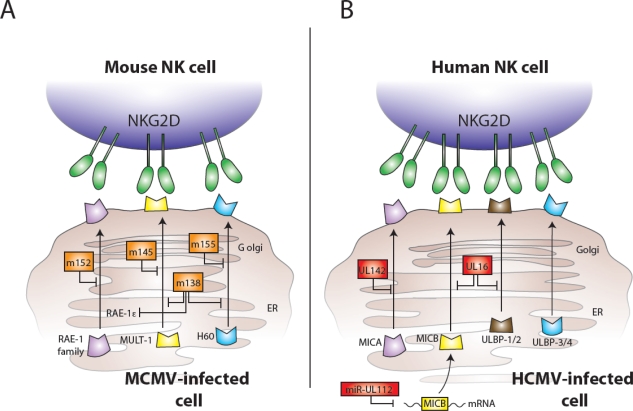
MCMV and HCMV proteins interfere with expression of NKG2D ligands and host recognition of virally infected cells. (**A**) MCMV-encoded glycoproteins (shown in orange) inhibit the expression of mouse NKG2D ligands: m152 interferes with expression of all five members of the RAE-1 family, m145 prevents surface expression of MULT1, m155 causes degradation of H60, and m138 assists to block expression of RAE-1ε, MULT1, and H60. (**B**) HCMV-encoded components (shown in red) also inhibit the expression of NKG2D ligands: UL142 inhibits MICA expression, UL16 binds MICB, ULBP1, and ULBP2 in the Golgi, and the miR-UL112 microRNA targets MICB mRNA for degradation leading to diminished cell surface expression of MICB.

During viral infection, another mechanism by which the host can communicate “danger” to the immune system is through the expression of stress molecules. In humans, these stress markers include the MIC and ULBP (also referred to as RAET1) family of molecules, which are ligands for the activating NKG2D receptor on NK cells and T cells [20–23]. In mice, the RAE-1 family of gene products, along with MULT1 and H60, constitute the stress-induced ligands of NKG2D, which are strongly induced during viral infection[24–27]. Although the mRNA transcripts of these NKG2D ligands are highly upregulated during infection, MCMV and HCMV have evolved gene products to limit the protein expression of NKG2D ligands. In MCMV, the viral protein m152, in addition to modulating MHC class I expression, interferes with expression of all five members of the RAE-1 family of glycoproteins [28], and the m145 and m155 proteins have been shown to downregulate MULT1 and H60 expression, respectively [29–31]. More recently, the viral protein m138 (also known as fcr-1) has been shown to play a redundant role, and is able to promiscuously suppress surface expression of NKG2D ligands MULT1, H60, and RAE-1ε [32,33]. Furthermore, m138 can also inhibit the expression of the costimulatory molecule CD80 (B7-1) on dendritic cells, leading to reduced CD8^+^ T cell priming against MCMV [34]. In HCMV, the viral glycoprotein UL16 downmodulates expression of the human NKG2D ligands MICB, ULBP1, and ULBP2 [23,35], and the virus-encoded UL142 shuts down expression of certain alleles of MICA to prevent NK cell recognition and killing [36,37]. More recently, Mandelboim and colleagues showed that HCMV also encodes a microRNA (named hcmv-miR-UL112) that specifically targets MICB transcripts for degradation, leading to decreased NKG2D binding and NK cell-mediated lysis of infected target cells[38]. These MCMV and HCMV-encoded proteins are depicted in Figure 2. Undoubtedly, additional virus-encoded proteins and microRNAs will be discovered that inhibit the ability of infected cells to signal the presence of the virus to NK cells and CD8^+^ T cells.

Both MCMV and HCMV possess genes encoding MHC class I-like molecules in their genomes. These homologs of MHC class I might serve as decoy ligands and allow the virus to hide from NK cells, which can sense the loss of MHC class I on potential target cells. Because inhibitory NK cell receptors, such as the mouse Ly49 receptors and the human KIRs, interact with MHC class I molecules, herpesviruses carrying homologues of class I might attempt to shut down the NK cell response.

NK cell-mediated resistance to MCMV in mice is genetically controlled. Previous genetic mapping studies showed that resistance to MCMV was restricted to the C57BL/6 and MA/My strains of mice [39–42]. The CMV-resistance gene(s), designated *Cmv1^r^*, is located on the distal end of mouse chromosome 6 [42–46], and this *Cmv1^r^* gene in C57BL/6 mice enables NK cells to protect mice from an otherwise lethal infection [41,47]. The precise molecular interactions between mouse NK cell receptors and viral elements on MCMV-infected cells have been elucidated, providing insights into the mechanisms behind NK cell-mediated anti-viral responses [48]. Similarly, a specific NK cell subset has been identified in humans that preferentially expands during HCMV infection, suggesting the parallel evolution of immune pathways in mouse and man that target members of the herpesvirus family.

## Ly49H and MCMV

**Figure 3.**
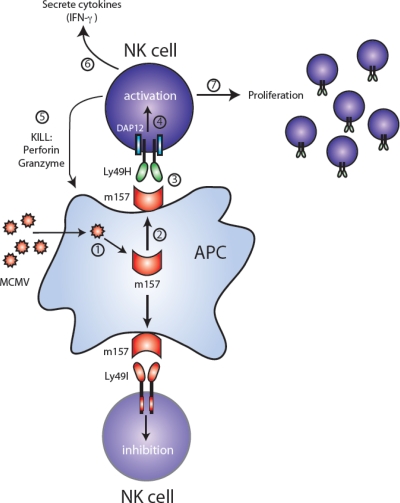
MCMV-encoded m157 leads to activation or inhibition depending on NK cell receptor expression. Following MCMV infection of a target cell (step 1), the m157 glycoprotein is expressed (step 2). NK cells from C57BL/6 mice express the activating Ly49H receptor (top half of figure), which associates with and signals through the ITAM-containing DAP12 adaptor molecule. Recognition of m157 by Ly49H-bearing NK cells (step 3) leads to activation via DAP12 (step 4), followed by cell-mediated cytotoxicity via perforin and granzymes (step 5), secretion of cytokines such as IFN-γ (step 6), and a clonal-like proliferation (step 7). In contrast, NK cells from 129/J mice express the ITIM-containing Ly49I receptor (bottom half of figure), which binds m157 on MCMV-infected cells, and become inhibited.

The NK cell response against MCMV constitutes arguably the most well-characterized example of a virus driving the evolution of immune receptors, and reciprocally the host immune system selecting for more virulent strains of a virus. In C57BL/6 mice, NK cells bearing the activating Ly49H receptor confer resistance against MCMV infection. Genetic linkage analysis, in conjunction with depletion of specific NK cell subsets by monoclonal antibodies, demonstrated that CMV-resistance is mediated by the Ly49H receptor, which is encoded by the *Cmv-1* locus [40,49,50]. The Ly49H receptor cannot signal on its own, but pairs via its transmembrane domain with an adapter molecule DAP12, which contains immunoreceptor tyrosine-based activation motifs (ITAMs) [51]. More recently, Ly49H has been shown to also pair with DAP10 [52–54], another adaptor molecule containing a YINM motif activating PI3-kinase and Vav1; signaling via DAP10 enhances DAP12-driven NK cell-mediated functions [53]. Thus, when Ly49H^+^ NK cells encounter MCMV-infected cells, the Ly49H receptor signals via DAP12 (augmented by DAP10), leading to NK cell activation, secretion of cytokines, and killing of the infected target cells (Figure 3). Not surprisingly, DAP12-deficient mice and DAP12 + DAP10 doubly-deficient mice are susceptible to MCMV infection, due to diminished expression of Ly49H and an inability to transmit a full activation signal [53,55]. Similarly, Ly49H-deficient mice on a C57BL/6 background, which possess NK cells unable to respond specifically against MCMV, are susceptible to viral infection [56,57]. Conversely, transgenic expression of Ly49H confers protection against MCMV in genetically susceptible BALB/c mice [58]. Thus, the presence of the Ly49H receptor (with its associated signaling subunits) is necessary and sufficient to provide NK cell-mediated resistance against MCMV infection.

The m157 glycoprotein encoded by MCMV was identified as a ligand of Ly49H using NFAT-GFP reporter cells expressing the Ly49H – DAP12 receptor complex [59,60]. Direct binding of m157 to Ly49H was further confirmed by using m157-Ig fusion proteins, which stained Ly49H transfectants and NK cells endogenously expressing Ly49H [59]. Additionally, Ly49H^+^ NK cells were able to mediate cytotoxicity and secrete IFN-γ when co-cultured with target cells expressing m157, and these effector functions were abrogated by a neutralizing anti-Ly49H antibody [59,60]. The expression of m157 in fibroblasts infected with MCMV is not dependent on β_2_-microglobulin (β_2_m) or TAP [59], demonstrating that m157 does not associate with MHC class I and is stably expressed on the surface of infected cells without a binding partner. The crystal structure of m157 has revealed homology to the nonclassical MHC class I family of molecules such as CD1d, H2-M3, and T22 [61]. When Ly49H-bearing NK cells detect m157 on MCMV-infected cells, this interaction leads to NK cell activation and effector function, followed by robust proliferation of Ly49H^+^ NK cells [62,63] (Figure 3). Not surprisingly, MCMV mutants lacking m157 are more virulent, resulting in higher viral titers in C57BL/6 mice following infection [64].

**Figure 4.**
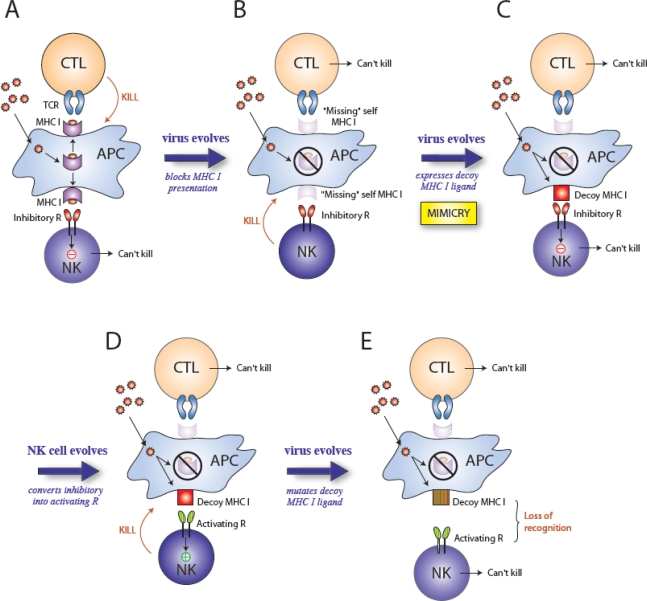
Selective pressures drive the evolution of virus and host genes. A model predicting the influence of natural selection on evolution of herpesviruses and NK cell receptors: (**A**) During infection, viral peptides presented on MHC class I are recognized by CD8^+^ T cells (CTL) and the infected cell is killed. (**B**) The virus evades the host immune response by evolving mechanisms to block MHC class I presentation. Although the CTL response is ineffective due to loss of antigen presentation, NK cells sense “missing self” (lack of MHC class I) and kill the infected cell because they are not inhibited. (**C**) The virus evades NK cell surveillance by the acquisition of a MHC class I decoy molecules (*i.e.* molecular mimicry), which bind inhibitory receptors on NK cells. Neither the CTL nor NK cell can kill the infected cell. (**D**) The host immune system evolves. NK cells acquire an activating receptor that recognizes the viral MHC class I decoy via duplication and gene conversion of the inhibitory receptor; NK cells now specifically recognize the viral component and kill the infected cell. (**E**) The virus evolves and continues to evade the host immune system, by mutating the decoy MHC class I molecule so that NK cells can no longer recognize or kill the infected cell.

The retention of a viral protein that specifically activates an immune response appears counterproductive to survival of the virus, so why would MCMV retain such a seemingly detrimental component in its genome? m157 is a MHC class I homolog; therefore, it may have evolved to engage inhibitory NK cell receptors and thus counteract viral proteins involved in blocking MHC class I expression leading to “missing self” recognition. Cytomegaloviruses possess many genes encoding immunoevasins that interfere with antigen presentation by preventing the assembly of MHC class I, or by retaining and degrading MHC class I (reviewed in [5,65]). Although inhibiting MHC class I presentation might protect the virus from a CD8^+^ T cell response (Figure 4A), the loss of MHC class I proteins on the surface of virus-infected cells could potentially lead to destruction by NK cells [66,67] (Figure 4B). Thus, downregulation of MHC class I by CMV might have forced the evolution of CMV-encoded class I-like homologs to engage inhibitory receptors on NK cells [68,69]. Thus, sensing of the viral MHC class I decoys that replace the vacated host MHC class I might suppress the NK cell response (Figure 4C). In certain strains of mice, for example 129/J, m157 binds the inhibitory Ly49I receptor [59], potentially impairing the ability of NK cells expressing Ly49I to respond to MCMV infection (Figure 3). Ly49I binds to m157 with higher affinity than with the Ly49H receptor [61]. Moreover, Ly49I binds to m157 with higher affinity than to the H2 ligand of Ly49I [61]. A comparison of the *Ly49i* and *Ly49h* genes indicates that *Ly49i* is evolutionary older and that *Ly49h* arose from a *Ly49i* gene by gene duplication and conversion [70]. During its evolution from Ly49I, Ly49H retained the ability to bind m157 (Figure 4D), but lost affinity for binding H2 ligands. Remarkably, due to selective pressure exerted by the virus, the host adapted [70–72]. Natural selection dictates that the virus will continue to mutate (Figure 4E), and this is evidenced by the high degree of polymorphism found in the *m157* gene from MCMV strains isolated from mice in the wild [73]. Furthermore, serial passage of MCMV in C57BL/6 mice leads to the escape of viral variants containing loss-of-function mutations in *m157* [73,74]. Together, these studies provide evidence that NK cells bearing specific activating receptors can exert ample selective pressure on a DNA virus, such that it undergoes rapid mutation at the molecular level in order to ensure its ability to evade immune surveillance and allow it to propagate.

## Ly49P and MCMV

Although MA/My mice do not possess the *Ly49h* gene, these mice are resistant to MCMV. Vidal and colleagues demonstrated that epistatic interactions between the activating NK cell receptor Ly49P and host MHC class I H2-D^k^ confer resistance to MCMV in MA/My mice [39], which possess both. Genetic mapping studies revealed that to resist MCMV infection mice must inherit both the *Ly49p* and *H2d^k^* genes from their parents [39]. Similar to the Ly49H receptor, Ly49P associates with and signals through DAP12; however, unlike m157 detection by Ly49H, the virus-derived ligand recognized by Ly49P is not as straightforward. NFAT-GFP reporter cells expressing Ly49P and DAP12 were activated only when co-cultured with MCMV-infected fibroblasts that expressed H2-D^k^ and recognition was blocked by a neutralizing anti-H2-D^k^ antibody, but not antibody to H2-K^k^ [39]. Thus, Ly49P recognition of MCMV is MHC-restricted, similar to MHC-restriction of T cells.

Recently, Vidal and colleagues identified a viral component required for Ly49P recognition of MCMV-infected cells. The virus-encoded m04 glycoprotein (also known as gp34) is crucial to NK cell-mediated control of viral replication, as a *m04* deletion mutant of MCMV abrogated resistance against infection in MA/My mice [75]. Thus, both H2-D^k^ and m04 are required for NK cell-mediated protection against MCMV infection. Using chimeric molecules of H2, where the α1, α2, and α3 domains of D^b^ and D^k^ were systematically exchanged, Vidal and colleagues demonstrated that the peptide-binding α1 and α2 domains of H2-D^k^ are absolutely required for recognition by Ly49P reporter cells [75]. However, Ly49P reporter cells were not activated when co-cultured with fibroblasts only expressing m04 and H2-D^k^ in the absence of MCMV infection [75]. Therefore, the ligand recognized by Ly49P appears to require additional elements conferred by MCMV infection. Together, these data suggest that unlike the ability of m157 to directly activate Ly49H^+^ NK cells, m04 is required but not sufficient for recognition by Ly49P. Interestingly, m04 associates with MHC class I proteins in MCMV-infected cells [7–9,76], and is able to inhibit antigen presentation without reducing the amount of MHC class I at the cell surface [77,78].

**Figure 5.**
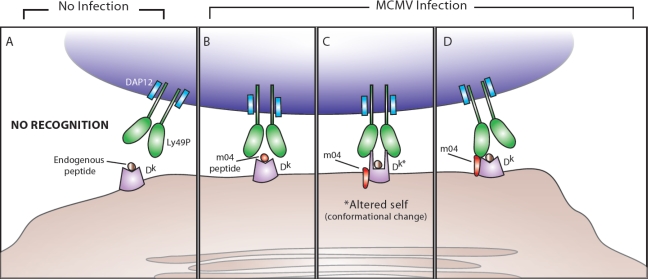
Possible mechanisms behind Ly49P recognition of m04 and H2-D^k^ during MCMV infection. (**A**) In the absence of MCMV infection, DAP12-associated Ly49P does not interact with MHC class I H2-D^k^ presenting endogenous peptides. (**B**) During MCMV infection, Ly49P-bearing NK cells might recognize a m04-derived peptide associated with H2-D^k^. (**C**) During MCMV infection, Ly49P might interact with a H2-D^k^ molecule that has undergone a conformational change because of m04 binding (the altered self-MHC class I is labeled in the diagram as Dk*). (**D**) During MCMV infection, Ly49P-bearing NK cells might recognize the entire m04/H2-D^k^ complex, with the extracellular domains of Ly49P interacting with portions of both m04 and H2-D^k^.

What is the nature of the ligand for Ly49P? Although prior studies using recombinant Ly49P demonstrated low affinity binding of Ly49P to H-2, Ly49P reporter cells do not recognize fibroblasts expressing H2-D^k^ with endogenous peptides [75] (Figure 5A). During MCMV infection, TAP, β_2_m, and the peptide binding groove (α1 and α2 domains) of H2-D^k^ are all required for Ly49P interaction, suggesting that Ly49P might recognize a peptide fragment of m04 bound to H2-D^k^ in manner similar to TCR recognition of peptide-MHC class I complexes (Figure 5B). Although this hypothesis remains plausible, the transfection of m04 into H2-D^k^-bearing fibroblasts without MCMV infection did not stimulate Ly49P reporter cells [75]. By interactions requiring its transmembrane domain, m04 has been shown to physically associate with β_2_m and MHC class I heavy chain in the endoplasmic reticulum prior to transport to the cell surface [8,9,76]. During MCMV infection, m04 could conceivably modify the conformation of H2-D^k^ so that Ly49P can recognize an altered form of H2-D^k^ (Figure 5C). Although expression of m04 in MCMV-infected fibroblasts does not appear to hinder recognition by CD8^+^ T cells (implying minimal allosteric changes in MHC class I) [79], NK cell recognition of a m04-associated H2-D^k^ protein could be entirely different, and does not exclude normal binding of peptides to the MHC class I groove or recognition of viral peptide-MHC by the T cell receptor. Alternatively, Ly49P might recognize the entire m04/H2-D^k^ complex, with the extracellular C-type lectin-like domains of Ly49P contacting both the intact viral m04 protein and the H2-D^k^ molecule (Figure 5D). Crystallization of the receptor bound to the ligand complex will reveal the nature of the viral ligand, as well as the specific amino acid residues on H2-D^k^ that are required both for m04 association and for Ly49P recognition during MCMV infection.

Similar to the extensive polymorphism observed in the *m157* gene found in MCMV isolates from wild mice, the high degree of variability of the *m04* amino acid sequence in multiple strains of MCMV suggests that mutation of this gene constitutes a mechanism by which the virus responds to selective pressures mediated by the host immune system [80]. Not surprisingly, the highest degree of polymorphism was found to reside in the extracellular domains of the m04 protein (where Ly49P might contact m04), whereas the transmembrane and cytoplasmic regions remained relatively conserved [80]. Selective pressure and consequent evolution of *m04* in MCMV strains from wild mice suggest that the host immune system can drive rapid mutation in specific viral proteins.

If the m04 protein confers recognition by Ly49P and enhances killing of infected cells, why does MCMV retain such a protein? Three possibilities exist. First, m04 might counteract the diminished cell surface expression of MHC class I mediated by the viral glycoproteins m152 and m06, which retain and mediate degradation of MHC class I proteins, respectively [7,10–12]. Because both m152 and m06 result in the loss of MHC class I on the cell surface, this might render MCMV-infected cells more susceptible to killing by NK cells able to sense “missing self”. Secondly, because m04 can selectively escort and stabilize MHC class I on the surface of infected cells, inhibitory NK cell receptors might gain more access to MHC class I, leading to suppression of NK cell responses. Several MHC class I molecules (including H2-K^b^ and H2-D^b^) have been shown to form complexes with m04 [8,9], and these same MHC class I molecules have been shown to be specific ligands for different inhibitory NK cell receptors, including Ly49A, Ly49C, Ly49I, Ly49J, and Ly49V [81–84]. Lastly, it has been recently proposed that the m04 regulation of antigen presentation may enhance adaptive immune responses leading to host survival [85]; the outcome, although favorable to the host, may benefit the virus as well, allowing MCMV to be disseminated within the population.

## CD94-NKG2C and HCMV

The first evidence that NK cells in humans were important for specific protection against cytomegalovirus came in 1989, when Biron and colleagues described a case of severe herpesvirus infection in a child lacking NK cells [86]. Unlike in the mouse models of cytomegalovirus infection described above, no specific viral ligands or NK cell receptors has yet been identified and characterized in HCMV. However, Lopez-Botet and colleagues have described the expansion of a specific subset of NK cells in response to HCMV. In the initial study, a substantial increase in the percentage of CD94-NKG2C receptor-expressing NK cells was observed in HCMV-seropositive individuals compared to control healthy adult blood donors [87]. One of the many difficulties of studying infectious diseases in humans is that it is hard to know when infection begins; therefore broadly characterizing immune cell subsets of HCMV-seropositive individuals only gives us snapshots of the immune response occurring days or months or even years after infection. Nonetheless, greater than 25% of the NK cells in HCMV-seropositive individuals expressed the activating NKG2C receptor, whereas the same subset of NK cells is rare (less than 2%) in seronegative subjects [87,88]. No such correlation between NK cell receptor expression and HCMV seropositivity was found for the related inhibitory NKG2A receptor [87,88]. Kuijpers and colleagues recently corroborated these results in a report describing preferential expansion of NKG2C^+^ NK cells in an immunodeficient child acutely infected with HCMV [89]. In this individual case, the time course of infection could be approximated and the peak of viremia closely correlated with the highly elevated NK cell numbers. Interestingly, the magnitude and kinetics of NK cell expansion and contraction closely resembles our recent measurements in the mouse Ly49H^+^ NK cell response following MCMV infection [63].

**Figure 6.**
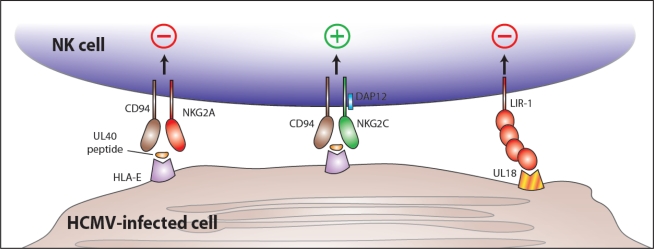
NK cell recognition of HCMV-encoded proteins during infection. The HCMV-encoded UL40 protein contains an epitope (from the UL40 leader sequence) that is bound by HLA-E and presented at the surface of virally infected cells. Binding of UL40 peptide/HLA-E by the inhibitory CD94/NKG2A receptor leading to NK cell inhibition could represent an immune evasion mechanism of HCMV. However, the activating CD94/NKG2C receptor might also engage the UL40 peptide/HLA-E complex (although with lower affinity) leading to NK cell activation. At the same time, the HCMV-encoded UL18 glycoprotein is expressed at the surface of infected cells and binds LIR-1, an inhibitory receptor, leading to inhibition of NK cell responses. (There is also evidence suggesting that the CD94/NKG2C may also recognize UL18, although a functional consequence for such an interaction is currently lacking.)

The human CD94-NKG2C receptor complex, expressed on NK cells and certain subsets of T cells, belongs to the killer C-type lectin-like receptor (KLR) family encoded in the NK cell gene complex on human chromosome 12 [46], and includes the NKG2A and NKG2C molecules which covalently associate with CD94 [90–94]. The CD94-NKG2A heterodimer is an inhibitory receptor that recruits the phosphatase SHP-1 via the immunoreceptor tyrosine-based inhibitory motif (ITIM) found in the cytoplasmic tail of NKG2A [95–98]. In contrast, the CD94-NKG2C heterodimer is activating, signaling via the associated ITAM-containing DAP12 subunit [99]. Both the inhibitory NKG2A and the activating NKG2C bind to a nonclassical MHC class Ib molecule HLA-E [100–102], which presents leader peptides derived from certain HLA-A, -B, -C, and -G proteins [100,103–105]. The affinity of NKG2A for HLA-E is 6-fold higher than the affinity of NKG2C. HLA-E is selectively resistant to downmodulation by the HCMV proteins that interfere with classical HLA class I expression. A leader segment from the UL40 HCMV protein can bind HLA-E, potentially inhibiting activation of CD94-NKG2A-bearing NK cells during HCMV infection [106–109]. Perhaps because of its restricted peptide-binding ability, HLA-E does not induce a robust CD8^+^ T cell response. Therefore, by selectively preserving expression of HLA-E, HCMV might avoid CTL detection, yet suppress NK cell-mediated responses (Figure 6).

NKG2C might represent the host’s response to CMV’s evasive maneuvers targeting NKG2A. Analysis of the genes encoding NKG2A and NKG2C in primates spanning greater than 35 million years of evolution suggests that both have been undergoing a “positive” selection indicative of host-pathogen struggles [110]. Is viral modulation of HLA-E enough to drive selection of a NKG2C receptor specific for HCMV?

The HCMV-encoded UL18 glycoprotein has structural homology to MHC class I, even associating with β_2_m [69,111]. UL18 binds with high affinity to leukocyte immunoglobulin-like receptor-1 (LIR-1) (also named LILRB1, CD85j, and ILT2), expressed in high amounts on monocytes and macrophages, B cells, and a subset of NK cells [112,113] (Figure 6). UL18 binds LIR-1 with a 3-log higher affinity than HLA class I [114]. Curiously, fibroblasts infected with a mutant HCMV lacking UL18 were found to be less susceptible to NK cell-mediated lysis than fibroblasts infected with wildtype strains of HCMV [115], suggesting that UL18 might enhance, rather than inhibit, NK cell activation. However, expansion of NKG2C^+^ NK cells *in vitro* was not abrogated when co-cultured with fibroblasts infected with mutant virus lacking UL18 compared to fibroblasts infected with strains containing UL18 [115]. Recent studies have demonstrated that NKG2C, but not NKG2A, weakly binds UL18 (K_D_ ∼10–100 μM) [110]. Nonetheless, as yet there is no direct evidence that NK cells expressing a CD94-NKG2C receptor directly recognize UL18 as a functional ligand.

## Concluding Remarks

Altogether, these findings show that immune surveillance by NK cells drives the selection of viral genes, and the ability of cytomegalovirus to reciprocally shape the NK cell receptor repertoire. Is this interplay between NK cells and CMV unique to CMV? As yet, there is no evidence that the NK cell receptors recognizing CMV, such as Ly49H or Ly49P, are involved in the control of any other viral pathogens. Reciprocally, viral proteins similar to m157 have not been detected, at least by computationally searching the genomes of any other pathogens. Unlike B cells and T cells that can generate new receptors by somatic gene recombination, NK cells possess only those receptors encoded by the germline. Therefore, selection of these receptors must act at the population level in the species, not within the individual. The remarkable diversity of the murine *Ly49* genes and primate *KIR* genes suggests an intriguing ongoing interaction between the host and pathogens. Perhaps the microbes of most interest to NK cells will be those pathogens, such as herpesvirus, that are persistent in the host and require constant vigilance.
